# Early developmental pathways to childhood symptoms of attention‐deficit hyperactivity disorder, anxiety and autism spectrum disorder

**DOI:** 10.1111/jcpp.12947

**Published:** 2018-07-02

**Authors:** Elizabeth Shephard, Rachael Bedford, Bosiljka Milosavljevic, Teodora Gliga, Emily J.H. Jones, Andrew Pickles, Mark H. Johnson, Tony Charman, The BASIS Team, Simon Baron‐Cohen, Patrick Bolton, Susie Chandler, Mayada Elsabbagh, Janice Fernandes, Holly Garwood, Kristelle Hudry, Greg Pasco, Leslie Tucker, Agnes Volein

**Affiliations:** ^1^ Department of Child and Adolescent Psychiatry Institute of Psychiatry, Psychology & Neuroscience King's College London London UK; ^2^ Biostatistics Department Institute of Psychiatry, Psychology & Neuroscience King's College London London UK; ^3^ Department of Psychology Institute of Psychiatry, Psychology & Neuroscience King's College London London UK; ^4^ Centre for Brain and Cognitive Development Birkbeck, University of London London UK; ^5^ South London and Maudsley NHS Foundation Trust London UK

**Keywords:** Autism spectrum disorder, attention‐deficit/hyperactivity disorder, anxiety, comorbidity, early developmental pathways

## Abstract

**Background:**

Children with autism spectrum disorder (ASD) often have co‐occurring symptoms of attention‐deficit/hyperactivity disorder (ADHD) and/or anxiety. It is unclear whether these disorders arise from shared or distinct developmental pathways. We explored this question by testing the specificity of early‐life (infant and toddler) predictors of mid‐childhood ADHD and anxiety symptoms compared to ASD symptoms.

**Methods:**

Infants (*n *=* *104) at high and low familial risk for ASD took part in research assessments at 7, 14, 24 and 38 months, and 7 years of age. Symptoms of ASD, ADHD and anxiety were measured by parent report at age 7. Activity levels and inhibitory control, also measured by parent report, in infancy and toddlerhood were used as early‐life predictors of ADHD symptoms. Fearfulness and shyness measured in infancy and toddlerhood were used as early‐life predictors of anxiety symptoms. Correlations and path analysis models tested associations between early‐life predictors and mid‐childhood ADHD and anxiety symptoms compared to mid‐childhood ASD symptoms, and the influence of controlling for ASD symptoms on those associations.

**Results:**

Increased activity levels and poor inhibitory control were correlated with ADHD symptoms and not ASD or anxiety; these associations were unchanged in path models controlling for risk‐group and ASD symptoms. Increased fearfulness and shyness were correlated with anxiety symptoms, but also ASD symptoms. When controlling for risk‐group in path analysis, the association between shyness and anxiety became nonsignificant, and when further controlling for ASD symptoms the association between fearfulness and anxiety became marginal.

**Conclusions:**

The specificity of early‐life predictors to ADHD symptoms suggests early developmental pathways to ADHD might be distinct from ASD. The overlap in early‐life predictors of anxiety and ASD suggests that these disorders are difficult to differentiate early in life, which could reflect the presence of common developmental pathways or convergence in early behavioural manifestations of these disorders.

## Introduction

Autism spectrum disorder (ASD) is a neurodevelopmental condition characterised by social communication deficits, restricted and repetitive behaviours and sensory atypicalities (DSM‐5; American Psychiatric Association, 2013). Many children with ASD also have symptoms of other neurodevelopmental disorders; two of the most frequently co‐occurring conditions in ASD are attention‐deficit/hyperactivity disorder (ADHD), characterised by developmentally inappropriate inattention, hyperactivity and impulsivity, and anxiety (Simonoff et al., 2008; Tick et al., 2016). Twin studies indicate shared genetic factors contribute to the frequent co‐occurrence of ADHD and anxiety in ASD (Hallett, Ronald, Rijsdijk, & Happé, 2010; Taylor et al., 2013; Tick et al., 2016), but beyond this, the reasons for the overlap between these disorders are not fully understood. In particular, it is unclear if ADHD and anxiety in ASD reflect the coexistence of distinct disorders with independent causal pathways. Alternatively, these disorders might arise from common causal pathways. For example, co‐occurring ADHD and anxiety symptoms could be a secondary consequence of ASD symptomatology, or ASD, ADHD, and anxiety could reflect different manifestations of the same underlying pathophysiological condition (Rommelse, Geurts, Franke, Buitelaar, & Hartman, 2011; Wood & Gadow, 2010). Distinguishing whether causal pathways are shared or distinct is important for understanding the nature of the co‐occurrence and aetiology of these three disorders.

Longitudinal analyses of causal associations between ASD and ADHD/anxiety traits across mid‐to‐late childhood have shown that ADHD traits more strongly predict later ASD traits than vice versa (Taylor et al., 2013; Tick et al., 2016), while ASD traits more strongly predict later anxiety traits than vice versa (Hallett et al., 2010; Tick et al., 2016). These findings suggest that ADHD symptoms are not simply a secondary consequence of ASD traits, while this may be the case for anxiety. Experimental studies in children have revealed separable neurocognitive abnormalities that are specifically associated with ASD and ADHD, and these ASD‐ and ADHD‐related abnormalities manifest in children with both disorders (Groom et al., 2017; Tye et al., 2014). These findings suggest that ASD and ADHD are dissociable in terms of brain function and that co‐occurring ASD and ADHD reflects the coexistence of two distinct disorders. In contrast, neurocognitive abnormalities associated with anxiety (without ASD) have been reported to be absent in children with ASD and co‐occurring anxiety (Hollocks, Ozsivadjian, Matthews, Howlin, & Simonoff, 2013; May, Cornish, & Rinehart, 2015), suggesting the neurocognitive basis of anxiety within ASD differs from anxiety outside of ASD, perhaps because anxiety is, in these cases, a secondary consequence of ASD symptoms.

One limitation of investigating the co‐occurrence of these disorders in childhood is that once symptoms are established it is difficult to disentangle whether they are caused by each other, independent pathophysiological mechanisms, compensatory processes, or the results of atypical developmental experiences (Johnson, Gliga, Jones, & Charman, 2015). An ideal method of investigating causal pathways leading to co‐occurring ADHD and anxiety symptoms in ASD is therefore to study their origins in infancy and track their development into childhood (Johnson et al., 2015). Prospective longitudinal studies have investigated the early (infancy to childhood) developmental pathways that lead to ASD, ADHD, and anxiety independently and have identified early‐life atypicalities that precede the onset of symptoms. For instance, difficulties disengaging visual attention, altered neurocognitive processing of social stimuli, and poor self‐regulation in infancy and toddlerhood predict later ASD symptoms (Jones, Gliga, Bedford, Charman, & Johnson, 2014; Szatmari et al., 2016). Increased locomotor activity and poor inhibitory control in infancy and toddlerhood and abnormal development of sustained visual attention (lack of improvement over time) predict higher childhood ADHD symptoms (Einziger et al., 2017; Miller, Iosif, Young, Hill, & Ozonoff, 2016; Willoughby, Gottfredson, & Stifter, 2017). High levels of infant and toddler fearfulness and shyness predict later anxiety symptoms (Gartstein et al., 2010; Prior, Smart, Sanson, & Oberklaid, 2000).

Few prospective longitudinal studies have explored early developmental pathways to these disorders simultaneously. In a population sample, Elberling et al. (2014) reported that abnormal activity (e.g. hyperactivity) and interests (e.g. lack of interest in play) in infancy predicted symptoms of both ASD and ADHD in early‐to‐mid childhood, suggesting these disorders might have similar origins in infancy and overlapping developmental pathways. However, activity and interests were combined in one variable, and it is possible that activity predicted ADHD while interests predicted ASD. Tonnsen, Malone, Hatton, and Roberts (2013) reported that increased infant and toddler fearfulness and reduced soothability were associated with early‐childhood anxiety symptoms but not ASD symptoms in children with Fragile X syndrome, suggesting early developmental pathways to anxiety might be separable from ASD. Drawing conclusions from these studies is complicated, however, as the analyses tested whether early‐life variables predicted each childhood disorder in separate models without controlling for symptoms of the other disorder/s.

In this study, we used longitudinal statistical modelling to test whether pathways to ADHD and anxiety were *shared with* or *distinct from* ASD. We tested, first, whether behavioural atypicalities in infancy and toddlerhood that have previously been associated with ADHD (increased locomotor activity, poor inhibitory control and sustained attention) were associated with mid‐childhood symptoms of ADHD but not ASD or anxiety, and whether atypicalities in infancy and toddlerhood that have previously been associated with anxiety (high fearfulness and shyness) were associated with mid‐childhood symptoms of anxiety but not ASD or ADHD. Second, we tested the specificity of those associations to ADHD and anxiety while controlling for the influence of ASD symptoms. We investigated this in a sample of infants at familial high‐risk for ASD (due to having an older sibling with ASD) studied prospectively into mid‐childhood. Around 20% of high‐risk infants develop ASD (Ozonoff et al., 2011) and another ~20% show other atypical developmental outcomes, including subclinical ASD traits, developmental delay, and symptoms of ADHD and anxiety (Miller, Iosif, Young, Hill, Phelps Hanzel et al., 2016; Ozonoff et al., 2014). Thus, our sample was defined based on risk for ASD but was also enriched for symptoms of ADHD and anxiety. We also included a sample of children at low‐risk for ASD studied prospectively alongside the high‐risk group and controlled for effects of risk‐group in analyses.

## Methods

### Participants

As part of the British Autism Study of Infant Siblings (BASIS; www.basisnetwork.org), 104 infants took part in research assessments at 7, 14, 24 and 38 months and were invited to return for a follow‐up study at age 7 years. At enrolment, each high‐risk (HR) infant (*n *=* *54) had an older sibling (in four cases, a half‐sibling) with a community clinical ASD diagnosis, confirmed using the *Development and Well‐Being Assessment* (*DAWBA*; Goodman, Ford, Richards, Gatward, & Meltzer, 2000) and the *Social Communication Questionnaire* (*SCQ*; Rutter, Bailey, & Lord, 2003) by expert clinicians on our team (TC, PB).^1^ Low‐risk (LR) controls (*n *=* *50) were full‐term infants (one exception) recruited from a volunteer database at the Birkbeck Centre for Brain and Cognitive Development. At enrolment, all LR infants had at least one older sibling; the SCQ was used to confirm absence of ASD in older siblings, with no child scoring above instrument cutoff (≥15, *n *=* *1 missing data). Not all children were retained at the 7‐year assessment (44 HR and 37 LR participated; see online Supporting Information for retention analysis). However, all 104 children were included in analyses and missing data were handled in statistical modelling using full information maximum likelihood (FIML; see Statistical Analysis below). Ethics approval was obtained from the NHS National Research Ethics Service (NHS RES London REC 08/H0718/76; 14/LO/0170). Parental written informed consent was obtained at all visits; child's written assent was obtained at the mid‐childhood visit.

### Measures

#### Mid‐childhood assessments

##### ADHD and anxiety

The parent‐rated *Conners 3* (Conners, 2008) was used to measure symptoms of ADHD (rated over the 6 months before testing). T‐scores (mean 50; *SD* 10; minimum‐maximum score ≤30 to ≥90) for the DSM‐IV‐TR (American Psychiatric Association, 2000) Inattention and Hyperactivity/Impulsivity domains were used in analyses. The parent‐report *Spence Children's Anxiety Scale* (*SCAS*; Spence, 1998) was used to assess current symptoms of anxiety. The SCAS is based on DSM‐IV‐TR (American Psychiatric Association, 2000) criteria for anxiety disorders. The Total Anxiety score (minimum‐maximum score 0–144) was used in analyses. None of the children had a clinical diagnosis of ADHD or anxiety according to parent report.

##### ASD

A battery of assessments was used to measure ASD symptoms and assign research diagnoses of ASD (see online Supporting Information for full details). Forty‐two of 44 HR and all 37 LR children completed the ASD assessments required for diagnostic evaluation in mid‐childhood. Of these, 15 HR children met DSM‐5 criteria for ASD and the remaining 27 HR and 37 LR children did not. A parent‐report measure of ASD symptoms (rated over the 6 months prior to testing), the *Social Responsiveness Scale – 2* (*SRS‐2*; Constantino, 2012), was used in the current analysis to be compatible with the parent‐report ADHD and anxiety measures. Age‐normed SRS‐2 T‐scores (mean 50; *SD* 10; minimum‐maximum ≤30 to ≥90) were used in analysis.

##### IQ

The *Wechsler Abbreviated Scale of Intelligence – Second Edition* (*WASI‐II*; Wechsler, 2011) was used to measure IQ. The age‐normed full‐scale intelligence quotient (FSIQ; mean 100, *SD* 15) was computed for each child.

#### Early‐life predictors of ADHD and anxiety

Early‐life behaviours predicted to be associated with later ADHD (locomotor activity, inhibitory control, sustained attention) and anxiety (fearfulness and shyness) were measured at the 7‐ and 14‐month visits using the Infant Behavior Questionnaire‐Revised (IBQ‐R, Gartstein & Rothbart, 2003) and at the 24‐month visit with the Early Child Behavior Questionnaire (ECBQ, Putnam, Gartstein, & Rothbart, 2006). The IBQ‐R (191 items) and ECBQ (201 items) are measures of temperament in which parents rate the frequency with which their infant/toddler exhibits particular behaviours in everyday contexts on a 7‐point scale (never to always). Parents rate their child's behaviour over the past week (IBQ‐R) or past 2 weeks (ECBQ). IBQ‐R/ECBQ items are averaged to yield subscales indexing different temperament dimensions, which show high continuity across instruments (Putnam, Rothbart, & Gartstein, 2008) and are therefore ideal for examining developmental pathways.

##### ADHD predictors

Locomotor activity was indexed by the IBQ‐R and ECBQ *Activity level* subscale at 7, 14 and 24 months. This subscale assesses the amount of limb movement, squirming and locomotor activity (IBQ‐R) and the rate and intensity of gross motor activity (ECBQ). Inhibitory control was indexed by the ECBQ *Inhibitory Control* subscale at 24 months (no IBQ‐R scale measures inhibitory control), which assesses the capacity to stop, moderate or refrain from behaviours under instruction. Sustained attention was indexed by the *Duration of Orienting* IBQ‐R subscale at 7 and 14 months, which measures attention to/interaction with an object for extended time periods, and the *Attentional Focusing* ECBQ subscale at 24 months, which measures sustained orienting to an object and resisting distraction. Example items for these scales are provided in the online Supporting Information. Higher scores reflect higher activity levels and better inhibitory control and attention.

##### Anxiety predictors

Fearfulness was indexed by the IBQ‐R and ECBQ *Fear* subscale at 7, 14 and 24 months. This subscale measures distress/startle to sudden environmental changes and inhibited approach to novelty (IBQ‐R), and startle to sudden events and negative affect related to anticipated pain, distress, and potentially threatening situations (ECBQ). Shyness was indexed by the ECBQ *Shyness* subscale at 24 months (no IBQ‐R subscale indexes shyness), which measures slow/inhibited approach or discomfort in novel/uncertain social situations. Example items are provided in the online Supporting Information. Higher scores reflect higher fearfulness and shyness.

### Statistical analysis

As a first step, we computed Pearson correlation coefficients between early‐life predictors and mid‐childhood symptoms of ADHD, anxiety, and ASD to examine how our hypothesised individual predictors and symptom domains were related to one another, before controlling for the influence of other variables and assessing the specificity of associations with ADHD and anxiety compared to ASD. ADHD, anxiety and ASD symptom scores were skewed and so an lnskew0 transformation was applied using Stata (StataCorp, 2013) prior to analysis, which normalised all scores (Shapiro–Wilk *p*‐values >.11). The α level was set to .05, with correlations considered statistically significant from zero if the *p* obtained was <.05. In addition, we highlight associations that remained significant with a Bonferroni‐corrected alpha level of .001 (α = .05/50 = .001), with correlations considered statistically significant if the obtained *p* was <.001. Early‐life variables that were not significantly associated with mid‐childhood symptoms in the Pearson correlations were not included in the next stage of analysis (longitudinal path analysis) to limit the number of variables entered into longitudinal models given our modest sample size.

In the next stage of analysis, we used path analysis run using Mplus (Muthén & Muthén, 1998) to test whether associations between early‐life predictors and mid‐childhood ADHD and anxiety symptoms were specific to those disorder symptoms (compared to ASD symptoms) and would hold while controlling for ASD symptoms. Missing data were considered missing at random and were analysed using full information maximum likelihood (FIML) methods (Enders & Bandalos, 2001). FIML is an effective method for analysing longitudinal data with missing data and has been demonstrated to provide less biased parameter estimates than listwise deletion (Enders, 2013; Widaman, 2006). Standardised model estimates (STDYX) are reported throughout. For predictors measured over multiple time‐points (activity level and fear), ‘Activity’ and ‘Fear’ factors were created by fixing the first factor loading with 7‐month activity level (or fear) at 1. This confirmatory factor analytic approach involves loading the measures, or indicator variables, from each of the three measurement occasions onto a single factor, essentially collapsing across time to give a weighted average, which accounts for measurement error. In Model 1a, these factors, together with risk‐group (high‐risk coded ‘1’ vs. low‐risk coded ‘0’), were used as predictors of mid‐childhood ADHD, anxiety and ASD symptoms to test whether Activity would be associated with later ADHD symptoms (and not ASD or anxiety) and Fear would be associated with later anxiety symptoms (and not ASD or ADHD). In Model 1b, mid‐childhood ASD symptoms were included as a predictor of ADHD and anxiety symptoms, rather than a correlated outcome, to assess whether controlling for ASD would affect associations between Activity and ADHD and Fear and anxiety.

Models 2a&b were saturated path analysis models assessing the association between inhibitory control and shyness (measured only at 24 months) and mid‐childhood ADHD, anxiety and ASD symptoms. Model 2a included inhibitory control, shyness and risk‐group as predictors of ADHD, anxiety and ASD symptoms to test whether inhibitory control would be associated with mid‐childhood ADHD symptoms (not ASD or anxiety) and shyness would be associated with mid‐childhood anxiety symptoms (and not ASD or ADHD). Model 2b included mid‐childhood ASD symptoms as a predictor rather than an outcome to assess whether controlling for ASD symptoms affected associations between inhibitory control and ADHD and shyness and anxiety.

We repeated these models including 1) ASD outcome rather than dimensional ASD symptoms as a covariate to assess whether the 15 HR children with ASD in mid‐childhood were driving effects; 2) sex and IQ as covariates to test whether these variables influenced associations; and 3) an interaction term between risk‐group and each early‐life predictor to check our assumption that associations between predictors and mid‐childhood symptoms would be similar in HR and LR groups. Due to space limitations, these additional models are reported and discussed in the online Supporting Information.

## Results

### Sample characteristics

Sample characteristics are presented in Table 1. The HR and LR groups did not differ in age or sex at any time‐point. The HR group had higher mid‐childhood hyperactivity/impulsivity, inattention, anxiety, and ASD scores, lower mid‐childhood IQ scores, and lower inhibitory control and higher shyness scores at 24 months than the LR group.

**Table 1:** Group characteristics and group means for symptoms in mid‐childhood (age 7 years) and early‐life predictor measures (age 7, 14, 24 months). Means (*SD*) by HR and LR group

	HR group	LR group	Group differences
7‐year measures			
Sex (*N* girls: *N* boys)	28:16	22:15	n/s
Age (months)	90.81 (6.33)	89.34 (4.81)	n/s
*N* (girls)	43 (27)	35 (21)	
Conners Inattention	57.07 (13.95)	51.22 (9.40)	*t* (72.30) = −2.21, *p *=* *.03, *d *=* *.49
*N* (girls)	42 (26)	37 (22)	
Conners Hyp/Imp	59.26 (16.59)	52.16 (11.58)	*t* (73.35) = −2.23, *p *=* *.03, *d *=* *.50
*N* (girls)	42 (26)	37 (22)	
SCAS Total Anxiety	21.16 (14.25)	12.22 (7.27)	*t* (77) = −3.41, *p *=* *.001^a^, *d *=* *.79
*N* (girls)	43 (27)	36 (22)	
SRS‐2 T‐score	59.27 (19.63)	45.49 (5.82)	*t* (42.62) = −4.07, *p *<* *.001^a^, *d *=* *.95
*N* (girls)	37 (23)	35 (21)	
WASI‐II FSIQ	109.34 (16.29)	117.06 (11.61)	*t* (74) = 2.34, *p *=* *.02, *d *=* *.55
*N* (girls)	41 (27)	35 (21)	
Transf. Inattention	3.02 (0.58)	2.77 (0.54)	
Transf. Hyp/Imp	2.87 (0.78)	2.55 (0.69)	
Transf. SCAS	2.87 (0.57)	2.31 (0.60)	
Transf. SRS‐2	2.66 (0.99)	1.81 (0.78)	
7‐month measures			
Sex (*N* girls: *N* boys)	33:21	29:21	n/s
Age (months)	7.31 (1.19)	7.38 (1.24)	n/s
*N* (girls)	54 (33)	50 (29)	
Activity level	4.19 (0.97)	4.28 (0.84)	n/s
*N* (girls)	52 (31)	49 (28)	
Orienting	3.41 (1.26)	3.63 (0.84)	n/s
*N* (girls)	52 (31)	49 (28)	
Fear	2.74 (1.16)	2.50 (0.94)	n/s
*N* (girls)	52 (31)	49 (28)	
14‐month measures			
Sex (*N* girls: *N* boys)	32:21	28:20	n/s
Age (months)	13.68 (1.57)	13.92 (1.33)	n/s
*N* (girls)	53 (32)	48 (28)	
Activity level	4.32 (0.92)	4.08 (0.71)	n/s
*N* (girls)	51 (30)	47 (27)	
Orienting	3.28 (1.03)	3.64 (0.92)	n/s
*N* (girls)	51 (30)	47 (27)	
Fear	3.33 (1.20)	2.95 (0.93)	n/s
*N* (girls)	51 (30)	47 (27)	
24‐month measures			
Sex (*N* girls: *N* boys)	32:20	27:20	n/s
Age (months)	23.92 (1.15)	23.87 (0.68)	n/s
*N* (girls)	53 (32)	47 (27)	
Activity level	4.86 (0.93)	4.72 (0.79)	n/s
*N* (girls)	46 (28)	47 (27)	
Attentional focus	4.12 (0.88)	4.28 (0.76)	n/s
*N* (girls)	46 (28)	47 (27)	
Fear	2.44 (1.01)	2.21 (0.53)	n/s
*N* (girls)	46 (28)	47 (27)	
Inhibitory control	3.53 (0.97)	4.03 (1.02)	*t* (91) = 2.40, *p *=* *.02, *d *=* *.50
*N* (girls)	46 (28)	47 (27)	
Shyness	3.41 (1.06)	2.82 (0.79)	*t* (91) = −3.07, *p *=* *.003, *d *=* *.63
*N* (girls)	46 (28)	47 (27)	

*N* (girls) *=* the number of participants in each group that completed each measure and the number of children completing the measure that were girls. Conners Inattention and Hyp/Imp = Conners 3 T‐scores for DSM‐IV Inattentive and Hyperactive/Impulsive domains. SCAS Total Anxiety = Spence Children's Anxiety Scale Total Anxiety score. SRS‐2 = Social Responsiveness Scale – 2. Transf. Inattention, Hyp/Imp, SCAS, SRS‐2 = Conners Inattention and Hyperactive/Impulsive T‐scores, SCAS Total Anxiety scores, and SRS‐2 T‐scores following lnkskew0 transformation. WASI‐II FSIQ = Wechsler Abbreviated Scale of Intelligence – 2nd Edition full‐scale IQ. Superscript ‘a’ indicates comparisons that remained significant after applying Bonferroni correction for multiple testing (α = .05/24 = .002). Note that corrected *df* and *p*‐values are reported wherever the assumption of equal variances was violated in *t*‐tests.

### Correlations between early‐life predictors and mid‐childhood ADHD, anxiety and ASD symptoms

Correlations between early‐life predictors and mid‐childhood ADHD, anxiety, and ASD symptoms are summarised in Table 2 and reported in the online Supporting Information. Inattentive symptoms were associated with higher activity levels and fear and lower inhibitory control at 24 months. Hyperactive/impulsive symptoms were associated with higher activity levels at all time‐points, higher fear at 24 months, and lower inhibitory control at 24 months. Anxiety symptoms were associated with higher fear at 14 and 24 months, higher activity levels at 14 months, and higher shyness at 24 months. ASD symptoms were associated with higher fear at 14 and 24 months, higher activity levels at 14 months, and higher shyness at 24 months. ADHD, anxiety and ASD symptoms were significantly positively associated with one another.

**Table 2:**
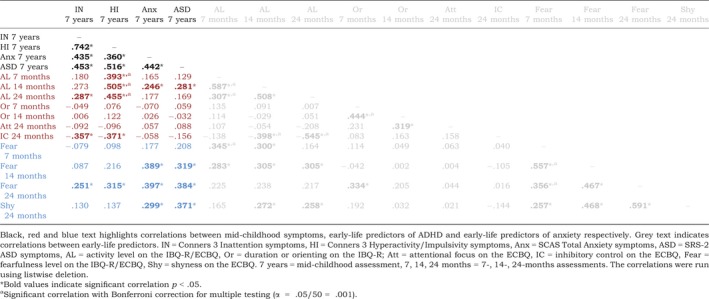
Correlation coefficients (Pearson *r*) for associations between early‐life predictor measures and mid‐childhood ADHD, anxiety and ASD symptoms in HR and LR groups combined [Colour table can be viewed at wileyonlinelibrary.com]

### Model 1a: Specificity of associations between early‐life activity level and fear and mid‐childhood ADHD and anxiety symptoms, respectively, compared to mid‐childhood ASD symptoms

**Figure 1:**
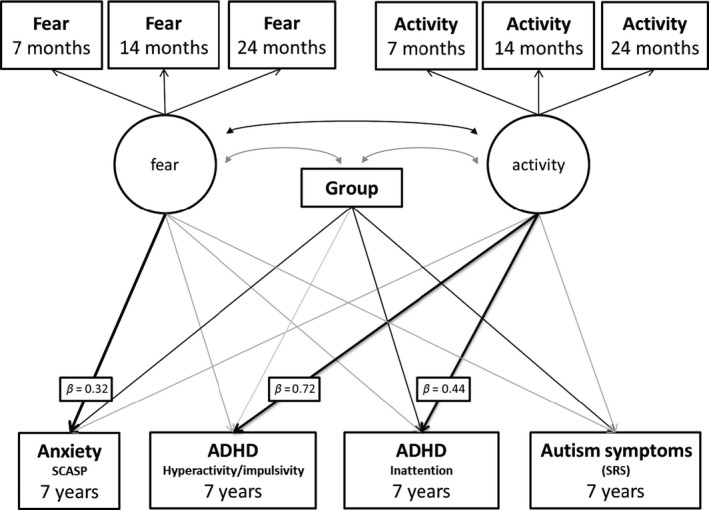
Model 1a: Path analysis model showing associations between infant Fear and Activity factors and later symptoms of ADHD, ASD and anxiety. Black arrows indicate significant effects at *p* < .05. Grey arrows are nonsignificant effects. Key pathways of interest are in bold with standardised beta values attached. Correlations between symptoms of ADHD, anxiety and ASD were included in the model but are not shown in the diagram for simplicity

A path analysis model (Figure 1) using a maximum likelihood estimator provided a good fit to the data (χ^2^(28) = 32.41, *p *=* *.26; comparative fit index, CFI = 0.99; root‐mean‐square error adjusted, RMSEA = 0.039). For the ‘Fear’ factor, all standardised factor loadings (SFL) were significant (7‐month SFL = 0.67, *p *<* *.001; 14 month SFL = 0.79, *p *<* *.001; 24 month SFL = 0.64, *p *<* *.001). Similarly, for the ‘Activity’ factor, factor loadings for all time‐points were significant (7‐month SFL = 0.69, *p *<* *.001; 14 month SFL = 0.81, *p *<* *.001; 24 month SFL = 0.61, *p *< .001). Fear and Activity were significantly positively correlated (*r *=* *.54, *p *<* *.001). Table 3 shows the standardised beta coefficients and corresponding *p‐*values for each path in the model. Activity was significantly associated with mid‐childhood hyperactivity/impulsivity and inattention, with higher activity levels across infancy and toddlerhood associated with higher ADHD symptoms. Activity was not significantly associated with ASD or anxiety. Fear was significantly associated with later anxiety, with higher infant and toddler fear associated with higher anxiety symptoms, but was not significantly associated with ASD or ADHD. Risk‐group was marginally associated with hyperactivity/impulsivity and significantly with inattention, anxiety and ASD, with higher symptoms in the high‐risk siblings.

**Table 3:** Summary of path analysis results. Standardised beta coefficients with their *p*‐values and 95% confidence intervals are shown for each path in Models 1a&b and 2a&b

	Standardised Beta (95% CI), *p* value
Model 1a	
Activity – Inattention	.44, (.12, .75), *p *=* *.006*
Activity – Hyp/Imp	.72, (.45, .99), *p *<* *.001*
Activity – Anxiety	.05, (−.24, .35), *p = *.72
Activity – ASD	.15, (−.17, .46), *p *=* *.37
Fear – Inattention	−.21, (−.54, .13), *p *=* *.22
Fear – Hyp/Imp	−.21, (−.52, .10), *p *=* *.19
Fear – Anxiety	.32, (.03, .62), *p *=* *.03*
Fear – ASD	.22, (−.09, .53), *p *=* *.16
Risk‐group – Inattention	.22, (.01, .44), *p *=* *.04*
Risk‐group – Hyp/Imp	.19, (−.004, .38), *p *=* *.06
Risk‐group – Anxiety	.36, (.17, .54), *p *<* *.001*
Risk‐group – ASD	.37, (.18, .56), *p *<* *.001*
Model 1b	
Activity – Inattention	.38, (.07, .68), *p *=* *.02*
Activity – Hyp/Imp	.66, (.39, .93), *p *<* *.001*
Activity – Anxiety	.03, (−.26, .32), *p *=* *.85
Fear – Inattention	−.30, (−.61, .02), *p *=* *.07
Fear – Hyp/Imp	−.29, (−.59, .00), *p *=* *.05
Fear – Anxiety	.28, (−.01, .58), *p *=* *.06
Risk‐group – Inattention	.08, (−.14, .29), *p *=* *.50
Risk‐group – Hyp/Imp	.04, (−.15, .23), *p *=* *.68
Risk‐group – Anxiety	.29, (.09, .49), *p *=* *.005*
ASD – Inattention	.41, (.18, .64), *p *=* *.001*
ASD – Hyp/Imp	.41, (.20, .62), *p *<* *.001*
ASD – Anxiety	.18, (−.05, .41), *p *=* *.12
Model 2a	
Inhibition – Inattention	−.33, (−.55, −.12), *p *=* *.002*
Inhibition – Hyp/Imp	−.35, (−.56, −.14), *p *=* *.001*
Inhibition – Anxiety	.06, (−.15, .28), *p *=* *.56
Inhibition – ASD	−.10, (−.33, .13), *p *=* *.41
Shyness – Inattention	.05, (−.17, .27), *p *=* *.65
Shyness – Hyp/Imp	.05, (−.16, .27), *p *=* *.62
Shyness – Anxiety	.16, (−.04, .37), *p *=* *.12
Shyness – ASD	.29, (.08, .50), *p *=* *.007*
Risk‐group – Inattention	.12, (−.10, .34), *p *=* *.29
Risk‐group – Hyp/Imp	.11, (−.12, .33), *p *=* *.35
Risk‐group – Anxiety	.40, (.20, .60), *p *<* *.001*
Risk‐group – ASD	.31, (.10, .52), *p *=* *.004*
Model 2b	
Inhibition – Inattention	−.29, (−.49, −.09), *p *=* *.005*
Inhibition – Hyp/Imp	−.30, (−.49, −.10), *p *=* *.003*
Inhibition – Anxiety	.09, (−.12, .30), *p *=* *.39
Shyness – Inattention	−.08, (−.29, .14), *p *=* *.49
Shyness – Hyp/Imp	−.10, (−.31, .11), *p *=* *.34
Shyness – Anxiety	.08, (−.13, .29), *p *=* *.47
Risk‐group – Inattention	−.01, (−.23, .21) *p *=* *.91
Risk‐group – Hyp/Imp	−.06, (−.26, .15), *p *=* *.57
Risk‐group – Anxiety	.31, (.10, .52), *p *=* *.004*
ASD – Inattention	.43, (.22, .64), *p *<* *.001*
ASD – Hyp/Imp	.53, (.34, .73), *p *<* *.001*
ASD – Anxiety	.29, (.07, .51), *p *=* *.01*

Significant paths in the models are indicated by asterisks. Activity = Activity factor reflecting IBQ‐R/ECBQ activity level scores across 7, 14, 24 months. Fear = Fear factor reflecting IBQ‐R/ECBQ fear scores across 7, 14, 24 months. Inattention = Conners 3 Inattention symptoms. Hyp/Imp = Conners 3 Hyperactive/Impulsive symptoms. ASD = SRS‐2 ASD symptoms. Anxiety = SCAS Total Anxiety symptoms. Risk‐group = high‐risk or low‐risk group. Inhibition = 24‐month inhibitory control scores on the ECBQ. Shyness = 24‐month shyness scores on the ECBQ.

### Model 1b: Associations between early‐life activity and fear and mid‐childhood ADHD and anxiety, respectively, controlling for ASD symptoms

In Model 1b, Model 1a was rerun controlling for ASD symptoms, rather than treating them as a correlated outcome. Model fit was good: χ^2^(28) = 32.53, *p *=* *.25, CFI = 0.98, RMSEA = 0.039. Factor loadings and the correlation between factors were unchanged from Model 1a. Activity remained a significant predictor of ADHD symptoms and not anxiety while controlling for ASD symptoms (Table 3). For Fear, the significant association with anxiety in Model 1a became marginal, with higher Fear marginally associated with higher anxiety. The nonsignificant association between Fear and ADHD in Model 1a also became marginally significant, with higher Fear associated with lower ADHD symptoms. ASD symptoms were significantly associated with higher hyperactive/impulsive and inattentive symptoms, but not with anxiety. The effect of risk‐group on hyperactivity/impulsivity and inattention became nonsignificant after accounting for ASD symptoms, but risk‐group remained a significant predictor of anxiety.

### Model 2a: Specificity of associations between early‐life inhibitory control and shyness and mid‐childhood ADHD and anxiety, respectively, compared to mid‐childhood ASD symptoms

**Figure 2:**
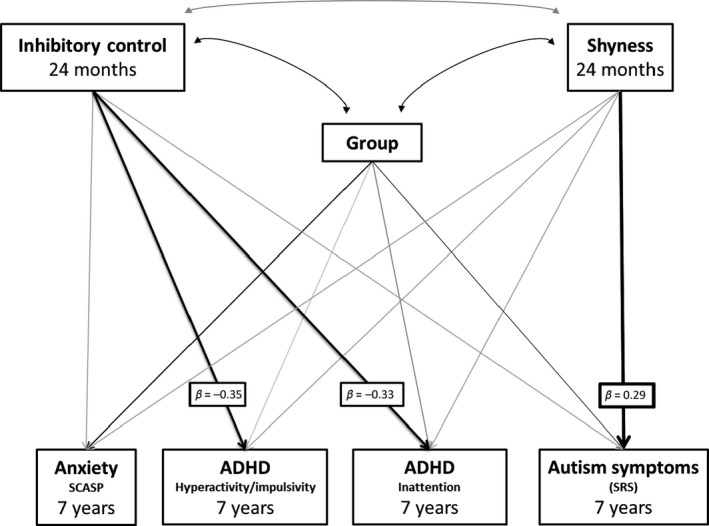
Model 2a: Path analysis model showing associations between 24‐month inhibitory control and shyness and later symptoms of ADHD, ASD and anxiety. Black arrows indicate significant effects at *p* < .05. Grey arrows are nonsignificant effects. Key pathways of interest are in bold with standardised beta values attached. Correlations between symptoms of ADHD, anxiety and ASD were included in the model but are not shown in the diagram for simplicity

A saturated path analysis model was run using maximum likelihood estimation to assess the association between 24‐month inhibitory control and shyness with mid‐childhood ADHD, anxiety and ASD symptoms, controlling for risk‐group (Figure 2). Inhibitory control and shyness were not significantly associated with each other (*r *=* *−.14, *p *=* *.162). Inhibitory control was significantly associated with inattentive and hyperactive/impulsive symptoms, with lower inhibitory control in toddlerhood associated with greater mid‐childhood ADHD symptoms, but was not significantly associated with ASD or anxiety (Table 3). Shyness was not significantly associated with mid‐childhood anxiety or ADHD symptoms, but higher shyness was associated with increased mid‐childhood ASD symptoms. Risk‐group was not significantly related to ADHD symptoms over and above shyness and inhibitory control, but was significantly related to anxiety and ASD, with higher symptoms in the high‐risk siblings.

### Model 2b: Associations between early‐life inhibitory control and shyness and mid‐childhood ADHD and anxiety, respectively, controlling for ASD symptoms

In Model 2b, we added ASD symptoms as a covariate, rather than a correlated outcome. Inhibitory control remained a significant predictor of ADHD symptoms and not anxiety while controlling for ASD symptoms (Table 3). Shyness did not significantly predict anxiety or ADHD. Risk‐group remained a significant predictor of anxiety, and not ADHD. ASD symptoms were significantly associated with higher levels of inattention, hyperactivity/impulsivity and anxiety.

### Interactions with risk‐group and effects of controlling for IQ, sex and ASD outcome

Results of additional models controlling for potential confounding variables are reported and discussed in full in the online Supporting Information. Risk‐group did not interact with early‐life predictor variables for our hypothesised pathways to ADHD or anxiety, though risk‐group did interact with shyness in predicting ADHD. Model results were not affected by covarying IQ. Controlling for ASD outcome produced similar findings to those reported in Models 1b&2b. Covarying for sex revealed a significant negative association between fearfulness and ADHD symptoms but did not affect our pathways of interest.

## Discussion

### Early developmental pathways to symptoms of ADHD compared to ASD symptoms

Increased early‐life locomotor activity and poor inhibitory control were correlated with mid‐childhood ADHD symptoms but (except 14‐month activity) not with ASD or anxiety. In path analysis, activity levels and inhibitory control were specifically associated with ADHD and not ASD or anxiety, and these associations remained when controlling for ASD symptoms and risk‐group. It has been argued that no neurocognitive or behavioural domain is impaired in ADHD and not in ASD and vice versa; consequently, these disorders must share pathophysiological mechanisms and represent different manifestations of the same underlying condition (Rommelse et al., 2011). Contrary to this view, our findings revealed early‐life atypicalities that were unique to ADHD symptoms and not shared by ASD, indicating these disorders may have distinct developmental origins and are therefore unlikely to reflect a common condition. Our findings are consistent with work in older children reporting dissociable neurocognitive abnormalities in ADHD and ASD (Groom et al., 2017; Tye et al., 2014) and longitudinal analyses suggesting that ADHD traits are not simply a consequence of ASD traits (Taylor et al., 2013; Tick et al., 2016). Our findings also replicate previously reported associations between increased activity levels and poor inhibitory control in infancy and toddlerhood and later ADHD symptoms (Einziger et al., 2017; Willoughby et al., 2017). We extend this work by showing these early‐life atypicalities specifically predict later ADHD and not ASD or anxiety within our sample.

In contrast to previous work (Miller, Iosif, Young, Hill, & Ozonoff, 2016), early‐life sustained attention was not associated with mid‐childhood ADHD symptoms. However, Miller, Iosif, Young, Hill, and Ozonoff (2016) used eye‐tracking to measure infants’ sustained attention to on‐screen stimuli while we used parent‐report ratings of infants’ ability to maintain attention during everyday activities. These methods provide complementary information about developing attentional abilities, and it is possible that eye‐tracking methods are more sensitive to early‐life atypicalities in sustained attention associated with later ADHD, while behaviourally observed difficulties with sustained attention emerge later in development.

### Early developmental pathways to symptoms of anxiety compared to ASD

Higher early‐life fearfulness and shyness were correlated with mid‐childhood anxiety symptoms, before controlling for ASD and risk‐group. However, fearfulness and shyness were also correlated with mid‐childhood ASD symptoms. Thus, early‐life atypicalities previously associated with anxiety (Gartstein et al., 2010; Prior et al., 2000) appeared not to be specific to anxiety, but rather were shared with ASD symptoms. In path analysis, fearfulness remained significantly predictive of anxiety when controlling for risk‐group but became marginal when controlling for ASD symptoms. Shyness did not predict anxiety when controlling for risk‐group or ASD, but did predict ASD when controlling for risk‐group. These findings indicate that at least part of the association between fear/shyness and anxiety detected in correlations was driven by ASD symptomatology. Our pattern of findings suggests that developmental pathways to anxiety can be difficult to differentiate from those leading to ASD, at least in our cohort.

Several possible reasons for the frequent co‐occurrence of anxiety in ASD have been proposed, including the coexistence of distinct disorders with independent causal pathways, an ASD‐specific variant of anxiety, and anxiety occurring as a consequence of ASD symptoms (e.g. social interaction difficulties leading to stress and anxiety) (Wood & Gadow, 2010). Our findings may be most in line with the last possibility: early‐life markers of anxiety (without ASD) were associated with later anxiety here, but were also associated with later ASD symptoms. However, it is also possible that our measures of fearfulness and shyness were not sufficiently sensitive to differentiate between early behavioural manifestations of anxiety and ASD. The IBQ‐R and ECBQ items assessing these constructs (see online Supporting Information for examples) could be endorsed for several reasons. High scores on Fear items may have indexed a generally fearful temperament associated with anxiety or early manifestations of ASD symptoms, such as aversions to changes in body placement and environment due to sensory issues or insistence on sameness. Items on the shyness scale may have detected early‐appearing social atypicalities associated with ASD, such as lack of interest in social interaction, rather than the socially inhibited temperament associated with anxiety. Future work should investigate other markers of anxiety in early life that are more clearly dissociable from ASD, such as physiological arousal measures which have been shown to differentiate between anxiety and ASD symptoms in older children (Chiu, Anagnostou, Brian, Chau, & Kushki, 2016).

### Clinical implications

High activity levels and poor inhibitory control in infancy and toddlerhood may be useful in the early detection and monitoring of developing ADHD symptoms. Early interventions designed to limit the development of ADHD symptoms may benefit from targeting the neurocognitive mechanisms that underlie inhibitory control and increased activity (Sonuga‐Barke & Halperin, 2010). Early‐life fearfulness and shyness have been proposed as risk‐markers for later‐emerging anxiety and have been targeted in early interventions (Kennedy, Rapee, & Edwards, 2009). Our findings indicate that these early‐life atypicalities may also detect ASD traits; consequently, interventions designed to improve early fearfulness and shyness in the development of anxiety may also affect early ASD traits.

Our findings also have implications for understanding the development of infants at familial high‐risk for ASD. It is known that these infants are vulnerable to anxiety, ADHD and other atypical developmental outcomes as well as ASD (Miller, Iosif, Young, Hill, Phelps Hanzel et al., 2016; Ozonoff et al., 2014), but few studies (Miller, Iosif, Young, Hill, & Ozonoff, 2016) have investigated early predictors of those outcomes. Our findings indicate that predictors of ADHD symptoms in these children can be detected in the first year of life. These predictors may be useful in monitoring the emergence of ADHD‐like problems in high‐risk infants. In contrast, our findings suggest that the early detection of anxiety is more complex in infants at high‐risk for ASD, and measures used as predictors of anxiety should be carefully selected to be distinguishable from early‐appearing ASD symptoms.

### Limitations and future directions

We used parent‐rated measures of early‐life predictors and mid‐childhood symptoms of ADHD, anxiety and ASD, which may have been subject to rater ‘halo effects’ compared to objective measures of early‐life predictors and multi‐informant or in‐depth clinical assessments of childhood symptoms. Our sample size was modest for longitudinal modelling, although the paths tested in our models were adequately powered. Post hoc power calculations using chi‐squared difference testing indicate good power to detect the three main hypothesised pathways (activity/inhibitory control – hyperactivity and inattention, and fear/shyness – anxiety), ranging between 71.7% and 99.8% across the four models. The sample size in the current study precluded a detailed psychometric approach using multigroup modelling to assess measurement invariance for the loading of items onto temperament subscales. This will be important in future studies to confirm whether the scales work in a similar way across risk groups. Our high‐risk sample was defined based on risk for ASD symptoms but not specifically for ADHD and anxiety, and a proportion of this sample met diagnostic criteria for ASD. These factors may have influenced our findings since ADHD and anxiety might manifest differently in the context of ASD/high‐risk for ASD. Still, our analysis including risk‐group, ASD symptoms, and ASD outcome as covariates indicated that our ADHD findings were robust to the influence of ASD and ASD‐risk, although this was not the case for anxiety. It will be important for future work to examine developmental pathways to these disorders in population samples and in multirisk group samples to assess the generalisability of our findings. We did not systematically assess the use of interventions in the high‐ or low‐risk groups and it is possible that some high‐risk children had received interventions for ASD or other developmental problems, which may have influenced developmental pathways. Risk for all three disorders was not evenly distributed across high‐ and low‐risk children, although we found no interactions between risk‐group and the early‐life predictors that were associated with ADHD and anxiety respectively. Finally, ASD, ADHD and anxiety tend to be diagnosed at different ages, with ASD in early childhood, ADHD in mid‐childhood, and anxiety in later childhood and adolescence (Beesdo, Knappe, & Pine, 2009; Christensen et al., 2016; Visser et al., 2014). Given the mid‐childhood age of our sample, it is possible that parent‐reports of anxiety were less reliable than those of ASD and ADHD, or that anxiety symptoms were not fully emerged in our sample.

## Conclusion

Our findings contribute to understanding the overlap between ADHD and anxiety symptoms in ASD. Co‐occurring ADHD and ASD might stem from distinct developmental pathways. In contrast, anxiety and ASD are difficult to differentiate early in life, perhaps because they result from shared early developmental pathways or due to convergence in early behavioural manifestations of these disorders.

**Key points**

- Attention‐deficit/hyperactivity disorder (ADHD) and anxiety commonly co‐occur in autism spectrum disorder (ASD), but whether these disorders arise from shared or distinct developmental pathways is unclear.
- Atypical infant and toddler activity levels and inhibitory control predicted mid‐childhood ADHD symptoms and not ASD, suggesting early developmental pathways to ADHD symptoms might be distinct from those that lead to ASD.
- Atypically high infant and toddler fearfulness and shyness predicted both mid‐childhood anxiety and ASD symptoms, suggesting developmental pathways leading to anxiety may be difficult to distinguish from those leading to ASD.
- Our findings indicate disorder‐specific early detection and intervention targets for ADHD and highlight that early markers for detecting and treating anxiety may also index ASD.

## Supporting information


**Appendix S1.** Participants: assessment of retention biases.
**Appendix S2.** Assessment of ASD symptoms and assignment of research diagnoses of ASD in mid‐childhood.
**Appendix S3.** Correlations between early‐life predictors and mid‐childhood ADHD, anxiety and ASD symptoms.
**Appendix S4.** Interactions with risk‐group in associations between early‐life predictors and mid‐childhood ADHD, anxiety and ASD symptoms.
**Appendix S5.** Interactions between risk‐group and early‐life activity level and fear in association with mid‐childhood ADHD, anxiety and ASD symptoms.
**Appendix S6.** Interactions between risk‐group and early‐life inhibitory control and shyness in association with mid‐childhood ADHD, anxiety and ASD symptoms.
**Table S1.** Example IBQ‐R and ECBQ subscale items.
**Table S2.** Correlation coefficients (Pearson *r*) for associations between early‐life predictor measures and mid‐childhood ADHD, anxiety and ASD symptoms excluding children with an ASD diagnosis (i.e. in LR and HR No ASD).

## Note

1 Five DAWBA and five SCQ missing.
