# Supplementary material for: Early developmental pathways to childhood symptoms of attention‐deficit hyperactivity disorder, anxiety and autism spectrum disorder
Source: J Child Psychol Psychiatry. 2018 Jul 2;60(9):963–74. doi: 10.1111/jcpp.12947 (PMC6694009; doi:10.1111/jcpp.12947)
Supplement: Supplementary file 1 — Appendix S1. Participants: assessment of retention biases. Appendix S2. Assessment of ASD symptoms and assignment of research diagnoses of ASD in mid‐childhood. Appendix S3. Correlations between early‐life predictors and mid‐childhood ADHD, anxiety and ASD symptoms. Appendix S4. Interactions with risk‐group in associations between early‐life predictors and mid‐childhood ADHD, anxiety and ASD symptoms. Appendix S5. Interactions between risk‐group and early‐life activity level and fear in association with mid‐childhood ADHD, anxiety and ASD symptoms. Appendix S6. Interactions between risk‐group and early‐life inhibitory control and shyness in association with mid‐childhood ADHD, anxiety and ASD symptoms. Table S1. Example IBQ‐R and ECBQ subscale items. Table S2. Correlation coefficients (Pearson r) for associations between early‐life predictor measures and mid‐childhood ADHD, anxiety and ASD symptoms excluding children with an ASD diagnosis (i.e. in LR and HR No ASD). [file JCPP-60-963-s001.docx]

**Additional Supporting Information for: Early developmental pathways to childhood symptoms of attention-deficit hyperactivity disorder (ADHD), anxiety, and autism spectrum disorder (ASD) – by Elizabeth Shephard et al.**

**Appendix S1.** Participants: assessment of retention biases.

Of the 104 infants (54 HR and 50 LR) initially recruited into the study in infancy, 53 HR (98%) and 48 LR (96%) children were retained up to age 3 years – the first time-point at which ASD outcomes were assigned and the last assessment time-point before the mid-childhood (age 7 years) follow-up study. Of the children retained to age 3 years, 44 HR (83%) and 37 LR (77%) were retained at the mid-childhood follow-up. The proportion of children retained between age 3 years and age 7 years did not differ between HR and LR groups (χ^2^ (1) = .844, *p* = .36). Possible retention biases were investigated by comparing symptoms of ASD, adaptive behaviour and developmental ability measured at age 3 years between the children retained to age 7 years and those not retained to age 7 years in the HR and LR groups separately. Age 3 ASD symptoms were assessed using the *Autism Diagnostic Observation Schedule – Second Edition* (*ADOS-2*; Lord et al., 2012), the *Social Communication Questionnaire (SCQ;* Rutter et al., 2003), and the *Social Responsiveness Scale – Second Edition* (*SRS-2*; Constantino, 2012); age 3 adaptive behaviour was assessed using the *Vineland Adaptive Behavior Scales – Second Edition, Vineland-II*; Sparrow et al., 2005) and developmental ability using the *Mullen Scales of Early Learning* (*MSEL;* Mullen, 1995). In the HR group, the retained children did not differ from the non-retained children in 3-year levels of ASD symptoms (all *p* > .52), adaptive functioning (*p* = .39), or developmental ability (*p* = .41). Similarly, the retained LR children did not differ from the non-retained LR children in 3-year ASD traits (all *p* > .85), adaptive functioning (*p* = .55) or developmental ability (*p* = .31).

**Appendix S2.** Assessment of ASD symptoms and assignment of research diagnoses of ASD in mid-childhood.

The *ADOS-2* (Lord et al., 2012), a standardised interaction observation assessment, was used to assess current symptoms of ASD (module 3 for 73 children, module 2 for one child, module 1 for one child; ADOS not completed with 3 LR children). The *Autism Diagnostic Interview – Revised* (*ADI-R*; Le Couteur et al., 2003), a structured parent interview, was completed with parents of HR children. Parents completed questionnaires to obtain further information on ASD symptoms and subclinical ASD traits. The *SRS-2* (Constantino, 2012) assessed parent-rated social impairments over the past 6 months. Higher scores on these measures reflect more severe ASD symptoms.

Experienced researchers who conducted the assessments (ES, BM, GP) and the lead clinician (TC) reviewed information on ASD symptomatology (ADOS-2, ADI-R (HR only), SCQ), adaptive functioning (measured by the *Vineland-II*), and IQ (measured by the *Wechsler Abbreviated Scale of Intelligence – Second Edition*, *WASI-II*; Wechsler, 2011) for each HR and LR child and as a team assigned clinical consensus best estimate diagnosis of ASD according to DSM-5 (American Psychiatric Association, 2013). Complete data on ASD symptomatology was obtained from 42 of the 44 high-risk children; of these, 15 (7 boys, 8 girls) met DSM-5 (APA, 2013) criteria for ASD and the remaining 27 children (8 boys, 19 girls) did not. None of the 37 low-risk children met DSM-5 criteria for ASD and none had a community clinical ASD diagnosis.

**Table S1.** Example IBQ-R and ECBQ subscale items.

Table S1 shows example items from the Infant Behavior Questionnaire – Revised (IBQ-R; Gartstein & Rothbart, 2003) and Early Child Behavior Questionnaire (ECBQ; Putnam, Gartstein & Rothbart, 2006) subscales used as early-life predictors of ADHD (Activity Level, Inhibitory Control, Duration of Orienting, Attentional Focus) and anxiety (Fear, Shyness).

| *IBQ-R/ECBQ subscale* | *Example item* |
| --- | --- |
| IBQ-R Activity Level | During feeding, how often did the baby squirm or kick? (Rated over the past week) |
| ECBQ Activity Level | While bathing, how often did your child splash, kick, or try to jump? (Rated over past two weeks) |
| ECBQ Inhibitory Control | When asked not to, how often did your child play with something anyway? (Rated over past two weeks) |
| IBQ-R Duration of Orienting | How often in the past week did the baby pay attention to your reading during most of the story when looking at picture books? |
| ECBQ Attentional Focus | When engaged in an activity requiring attention, such as building blocks, how often did your child stay involved for 10 minutes or more (Rated over past two weeks) |
| IBQ-R Fear | How often in the last week did the baby cry or show distress at a parents’ change in appearance (glasses off, shower cap on, etc.)? |
| ECBQ Fear | While at home, how often did your child show fear at a loud sound (blender, vacuum cleaner, etc.)? (Rated over past two weeks) |
| ECBQ Shyness | In situations when s/he is meeting new people, how often did your child become quiet? (Rated over past two weeks) |

**Appendix S3.** Correlations between early-life predictors and mid-childhood ADHD, anxiety, and ASD symptoms.

Inattentive symptoms were significantly associated with higher activity levels at 24m (*r*(73) = .287, *p =* .01), higher fear at 24m (*r*(73) = .251, *p* = .03), and lower inhibitory control at 24m (*r*(73) = -.357, *p =* .002). Hyperactive/impulsive symptoms were significantly associated with higher activity levels at 7m (*r*(78) = .393, *p <* .001), 14m (*r*(76) = .505, *p <* .001), and 24m (*r*(73) = .455, *p <* .001), higher fear at 24m (*r*(73) = .315, *p =* .007), and lower inhibitory control at 24m (*r*(73) = -.371, *p =* .001). Anxiety symptoms were significantly associated with higher activity levels at 14m (*r*(76) = .246, *p* = .03), higher fear at 14m (*r*(76) = .389, *p =* .001) and 24m (*r*(73) = .397, *p =* .001) and higher shyness at 24m (*r*(73) = .299, *p =* .01). ASD symptoms were associated with higher activity levels at 14m (*r*(69) = .281, *p* = .02), higher fear at 14m (*r*(69) = .319, *p* = .008) and 24m (*r*(66) = .384, *p =* .001) and higher 24m shyness (*r*(66) = .371, *p* = .002). The associations between activity levels in infancy and toddlerhood and mid-childhood hyperactive/impulsive symptoms remained significant when applying a Bonferroni correction for multiple comparisons (α = .05/50 = .001), while the remaining associations did not.

*Including additional model covariates:*

*Covarying for categorical ASD outcome:* In Models 1b and 2b in the main paper we covaried for ASD symptoms and risk group. Here, we test whether the results remain similar when controlling instead for categorical ASD outcome. To do this we entered dummy variables: dummy 1 (D1) comparing ASD with low-risk group and dummy 2 (D2) comparing high-risk no ASD with low-risk controls.

For Model 1b, the model remained a good fit to the data (χ^2^(28) = 32.81, *p* = 0.24, comparative fit index; CFI = 0.98, root mean square error adjusted; RMSEA = 0.041) and results remained similar, with activity significantly predicting inattention (β = 0.39, *p* = 0.016) and hyperactivity (β = 0.67, *p* > 0.001) but not anxiety (*p* = 0.76). However, the previously marginal effect of fear on anxiety became significant (β = 0.34, *p* = 0.023), and the marginal effects of fear on inattention and hyperactivity became non-significant (*p* values > 0.20). D1 (ASD vs. low-risk) was a significant predictor of inattention (β = 0.26, *p* = 0.017), hyperactivity (β = 0.23, p = 0.023) and anxiety (β = 0.27, *p* = 0.009), whereas D2 (high-risk no ASD vs. low-risk) was a significant predictor of only anxiety (β = 0.32, *p* = 0.001), not inattention or hyperactivity (*p* values > 0.083).

For Model 2b, the significance level of the effects remained unchanged when controlling for dummy variables for ASD outcome rather than continuous ASD symptoms and risk group. Inhibitory control remained a significant negative predictor of inattention and hyperactivity (β = -0.31, *p* = 0.006; β = -0.30, *p* = 0.006 respectively) and not anxiety (*p* = 0.48). The association between shyness and inattention, hyperactivity and anxiety remained non-significant (*p* values > 0.22). D1 (ASD vs. low-risk) was significantly associated with hyperactivity (β = 0.32, *p* = 0.009) and anxiety (β = 0.34, p = 0.004), but only marginally with inattention (β = 0.24, *p* = 0.059). For D2 (high-risk no ASD vs. low-risk) only the association with later anxiety traits was significant (β = 0.35, *p* = 0.001). The association between D2 and inattention and hyperactivity were not significant (*p* values > 0.38).

These results are similar to those reported in Models 1a and 2a, which control for risk group, but not symptoms of ASD, suggesting that the results are not being driven by the subset of children with an ASD diagnosis. To further illustrate this, we present correlations between early-life predictors and mid-childhood symptoms after removing the subset of children with an ASD diagnosis (see Table S2). The correlations show that associations between early-life predictors and mid-childhood symptoms of ADHD, anxiety and ASD were similar when children with ASD were removed.

**Table S2.** Correlation coefficients (Pearson *r*) for associations between early-life predictor measures and mid-childhood ADHD, anxiety, and ASD symptoms excluding children with an ASD diagnosis (i.e. in LR and HR No ASD).

|  | *IN*  *7yr* | *HI*  *7yr* | *Anx 7yr* | *ASD 7yr* | *AL*  *fs* | *IC*  *24m* | *Fear*  *fs* | *Shy 24m* |
| --- | --- | --- | --- | --- | --- | --- | --- | --- |
| **Non-ASD (LR & HR-non-ASD (*N* = 73^a^)** | | | | | | | | |
| *IN 7yr* | ---- |  |  |  |  |  |  |  |
| *HI 7yr* | .709*^B^ | ---- |  |  |  |  |  |  |
| *Anx 7yr* | .342* | .225 | ---- |  |  |  |  |  |
| *ASD 7yr* | .356* | .389* | .338* | ---- |  |  |  |  |
| *AL fs* | .346* | .658*^B^ | .220 | .343 | ---- |  |  |  |
| *IC 24m* | -.357* | -.358* | -.126 | -.176 | -.453*^B^ | ---- |  |  |
| *Fear fs* | -.072 | .116 | .379* | .330* | .560*^B^ | -.254* | ---- |  |
| *Shy 24m* | -.086 | -.219 | .125 | .200 | .038 | -.155 | .453*^B^ | ---- |

* significant correlation *p* < .05. ^B^ significant correlation following Bonferroni correction for multiple tests (α = .05/50 = .001). Black, red, and blue text highlights correlations between mid-childhood symptoms, early-life predictors of ADHD, and early-life predictors of anxiety, respectively. *IN* = Conners 3 Inattention symptoms, *HI* = Conners 3 Hyperactivity/Impulsivity symptoms, *Anx* = SCAS Total Anxiety symptoms, *ASD* = SRS-2 ASD symptoms, *AL* fs= activity level factor score from the IBQ-R/ECBQ, *IC* = inhibitory control on the ECBQ at 24m, Fearfulness factor score from the IBQ-R/ECBQ, *Shy* = shyness on the ECBQ at 24m. The correlations were run using listwise deletion. Scores for symptoms of ADHD, anxiety and ASD were transformed using a lnkskew0 transformation prior to computing correlation coefficients.

*Covarying for sex:* Model 1a provided a good fit to the data after adding sex as a covariate (χ^2^(32) = 33.56, *p* = 0.39, comparative fit index; CFI = 0.995, root mean square error adjusted; RMSEA = 0.022). Activity levels remained significantly associated with hyperactivity and inattention (β = 0.85, *p* < 0.001 and β = 0.54, *p* = 0.001 respectively) and were not significantly associated with anxiety (β = 0.10, *p* = 0.53) or ASD traits (β = 0.22, *p* = 0.17). The effect of fear on inattention and ASD traits remained non-significant (*p* values > 0.11). The effect of fear on hyperactivity, which was previously non-significant, became marginal after covarying for sex (β = -0.30, *p* = 0.059) and the effect on anxiety, which was previously significant, became marginal (β = 0.30, *p* = 0.051). Sex was a significant predictor of hyperactivity (β = 0.41, *p* < 0.001), inattention (β = 0.31, *p* = 0.004) and ASD traits (β = 0.26, *p* = 0.012), with higher traits in girls, but not anxiety (β = 0.17, *p* = 0.084).

Model 1b also remained a good fit to the data after covarying for sex (χ^2^(32) = 33.56, *p* = 0.39, comparative fit index; CFI = 0.995, root mean square error adjusted; RMSEA = 0.022). For activity, the results remained the same, with a significant effect for hyperactivity (β = 0.78, *p* < 0.001) and inattention (β = 0.46, *p* = 0.006) but not anxiety (*p* = 0.67). For fear, the effect on anxiety remained marginal (β = 0.28, *p* = 0.07), but the marginal negative effects with inattention and hyperactivity became significant (β = -0.33, *p* = 0.046; β = -0.35, *p* = 0.025 respectively). Sex was a significant predictor of hyperactivity (β = 0.33, *p* = 0.001) and inattention (β = 0.22, *p* = 0.043), with higher traits in girls, but not significantly associated with anxiety (*p* = 0.185).

The positive associations that emerged between fear and ADHD symptoms when controlling for sex were unexpected and in contrast to previous research reporting associations between lower fearfulness (‘fearlessness’) in toddlerhood and higher ADHD symptoms at school-age (Pappa et al., 2014). We further investigated our effects by examining correlations between the fear factor and ADHD symptoms in boys and girls separately. We found a significant positive association between fear and hyperactive/impulsive symptoms in girls (*r*(48) = 0.328, *p* = 0.02), but no significant associations between fear and ADHD symptoms in boys (both *r* < .235, both *p* > .20). It is possible that for girls, early temperamental fearfulness is a risk factor for a range of developmental problems, while in boys, early fearfulness is more specifically associated with later anxiety and/or ASD.

For Models 2a and 2b covarying for sex had no impact on the significance level of the results.

*Covarying for IQ:* For all models, the significance level of results remained the same after covarying for Wechsler Abbreviated Scale of Intelligence – Second Edition, full-scale IQ measured at the 7-year visit.

**Appendix S4.** Interactions with risk-group in associations between early-life predictors and mid-childhood ADHD, anxiety, and ASD symptoms.

Our main analyses examining longitudinal associations between early-life predictors (activity level, inhibitory control, orienting/attentional focus, fear, shyness) and mid-childhood symptoms of ADHD, anxiety, and ASD (Models 1a&b and 2a&b) were conducted in the high-risk and low-risk groups combined since we assumed early developmental pathways to symptoms of ADHD and anxiety would manifest similarly in children with and without familial risk for ASD. We included risk-group as a variable in those models to control for any influence of familial risk for ASD on predictive associations. In the current supplementary analyses we tested our assumption more explicitly by examining interactive effects between early-life predictors and risk-group in associations with mid-childhood symptoms. Inhibitory control and shyness variables were mean centred, before interactions were computed.

**Appendix S5.** Interactions between risk-group and early-life activity level and fear in association with mid-childhood ADHD, anxiety, and ASD symptoms.

For Models 1a and 1b, we added interactions between risk group and the latent factors Activity and Fear via the XWITH option in Mplus (Muthén & Muthén, 1998-2012). Only unstandardized coefficients are available with XWITH, type=random option. Models were run using maximum likelihood with robust standard errors (MLR), to examine interactions between the Activity and Fear factors and risk-group in association with later symptoms of ADHD, anxiety, and ASD (Model 1a, BIC = 2139.13) or in association with later symptoms of ADHD and anxiety while controlling for ASD symptoms (Model 1b, BIC = 1484.48). There were no significant interactions between risk-group and Activity or Fear in associations with ADHD, anxiety, or ASD symptoms (*p* values > 0.19), or between ADHD and anxiety while controlling for ASD symptoms (*p* values > 0.17). These findings indicate that associations between early-life activity levels and fear and mid-childhood symptoms of ADHD, anxiety, and ASD were similar in high-risk and low-risk groups.

**Appendix S6.** Interactions between risk-group and early-life inhibitory control and shyness in association with mid-childhood ADHD, anxiety, and ASD symptoms.

For Models 2a&b, interactions were added between 24m inhibitory control and shyness and risk-group in association with later symptoms of ADHD, anxiety, and ASD (Model 2a) or in association with later symptoms of ADHD and anxiety while controlling for ASD symptoms (Model 2b). Models were run using maximum likelihood estimation. Interactions between risk-group and inhibitory control in association with ADHD, anxiety, and ASD symptoms were non-significant (*p* values > 0.07), indicating the associations between inhibition and mid-childhood symptoms were similar in high- and low-risk groups. Similarly, the interaction between risk-group and shyness in association with inattention, anxiety and ASD symptoms were non-significant (*p* values > 0.47). However, the interactions between risk-group and shyness in association with hyperactivity/impulsivity were significant with and without controlling for ASD symptoms (Model 2a β = 0.36, *p* = 0.03 and Model 2b β = 0.91, *p* = 0.01). To further investigate these interactions, we computed Pearson correlation coefficients between shyness and hyperactive/impulsive symptoms in the high-risk and low-risk groups separately. In the high-risk group, this association was positive and reached trend-level (*r*(37) = .280, *p* = .09, *r^2^* = .08), while in the low-risk group the association was negative and non-significant (*r*(36) = -.235, *p* = .17, *r^2^* = .06). These findings suggest that children at HR and LR for ASD may show different associations between ADHD and shyness, with HR children showing a positive relationship (more shyness, higher ADHD symptoms) and the LR children showing a negative relationship (more shyness, less ADHD). The negative direction of the association in the low-risk group is consistent with reports of positive associations between Surgency, a temperament factor similar to extraversion (the opposite of shyness), and later ADHD symptoms (Martel et al., 2014). Concerning the positive association in the high-risk group, it is possible that shyness in this group indexed early ASD-like traits which were over-represented in this group and which in turn were associated with later ADHD symptoms. This interpretation is supported by the strong positive correlation between ASD and ADHD symptoms in mid-childhood (see main text Table 2).

**References**

American Psychiatric Association. (2013). *Diagnostic and Statistical Manual of Mental Disorders (DSM-5)*. Washington DC: American Psychiatric Association.

Constantino, J.N. (2012). *Social Responsiveness Scale, Second Edition (SRS-2).* Los Angeles, CA: Western Psychological Services.

Gardiner, E., & Iarocci, G. (2018). Everyday executive function predicts adaptive and internalizing behavior among children with and without autism spectrum disorder. *Autism Research*, *11*(2), 284-295.

Gartstein, M. A., & Rothbart, M. K. (2003). Studying infant temperament via the revised infant behavior questionnaire. *Infant Behavior and Development*, *26*(1), 64-86.

Le Couteur, A., Lord, C., & Rutter, M. (2003). *The Autism Diagnostic Interview-Revised (ADI-R).* Los Angeles, CA: Western Psychological Services.

Lord, C., Rutter, M., DiLavore, P. C., Risi, S., Gotham, K., & Bishop, S. L. (2012). *Autism Diagnostic Observation Schedule: ADOS-2*. Los Angeles, CA: Western Psychological Services.

Martel, M. M., Gremillion, M. L., Roberts, B. A., Zastrow, B. L., & Tackett, J. L. (2014). Longitudinal prediction of the one-year course of preschool ADHD symptoms: Implications for models of temperament–ADHD associations. *Personality and individual differences*, *64*, 58-61.

Mullen, E. M. (1995). *Mullen scales of early learning*. Circle Pines, MN: American Guidance Service.

Muthén, L.K. and Muthén, B.O. (1998-2012). *Mplus User’s Guide. Seventh Edition*. Los Angeles, CA: Muthén & Muthén.

Pappa, I., Mileva-Seitz, V. R., Szekely, E., Verhulst, F. C., Bakermans-Kranenburg, M. J., Jaddoe, V. W., ... & van IJzendoorn, M. H. (2014). DRD4 VNTRs, observed stranger fear in preschoolers and later ADHD symptoms. *Psychiatry research*, *220*(3), 982-986.

Putnam, S. P., Gartstein, M. A., & Rothbart, M. K. (2006). Measurement of fine-grained aspects of toddler temperament: The Early Childhood Behavior Questionnaire. *Infant Behavior and Development*, *29*(3), 386-401.

Rutter, M., Bailey, A., & Lord, C. (2003). *The social communication questionnaire: Manual*. Los Angeles, CA: Western Psychological Services.

Sparrow, S. S., Balla, D. A., & Cicchetti, D. V. (2005). *Vineland Adaptive Behavior Scales, Second Edition (Vineland-II).* Bloomington, NM: Pearson Assessments.

Wallace, G. L., Kenworthy, L., Pugliese, C. E., Popal, H. S., White, E. I., Brodsky, E., & Martin, A. (2016). Real-world executive functions in adults with autism spectrum disorder: Profiles of impairment and associations with adaptive functioning and co-morbid anxiety and depression. *Journal of autism and developmental disorders*, *46*(3), 1071-1083.

Wechsler, D. (2011). *WASI-II: Wechsler abbreviated scale of intelligence*. New York, NY: Psychological Corporation.

White, S. W., Oswald, D., Ollendick, T., & Scahill, L. (2009). Anxiety in children and adolescents with autism spectrum disorders. *Clinical psychology review*, *29*(3), 216-229.
